# [Corrigendum] Role of Thrombospondin‑1 in sepsis‑induced myocardial injury

**DOI:** 10.3892/mmr.2023.13000

**Published:** 2023-04-20

**Authors:** Yun Xie, Jiaxiang Zhang, Wei Jin, Rui Tian, Ruilan Wang

Mol Med Rep 24: 869, 2021; DOI: 10.3892/mmr.2021.12509

Subsequently to the publication of this paper, the authors’ noticed that the same β-actin control bands were inadvertently used in the western blots shown in Figs. 1A and 2A. After having examined their original data, the authors realized the control bands were chosen incorrectly for Fig. 1, but were able to identify the data that should have been used for this figure.

The revised version of Fig. 1, showing the correct western blotting data for Fig. 1A, is shown opposite. Note that this error did not significantly affect either the results or the conclusions reported in this paper, and all the authors agree to the corrigendum. Furthermore, the authors thank the Editor of *Molecular Medicine Reports* for allowing them the opportunity to publish this corrigendum, and apologize to the readership for any inconvenience caused.

## Figures and Tables

**Figure 1.**
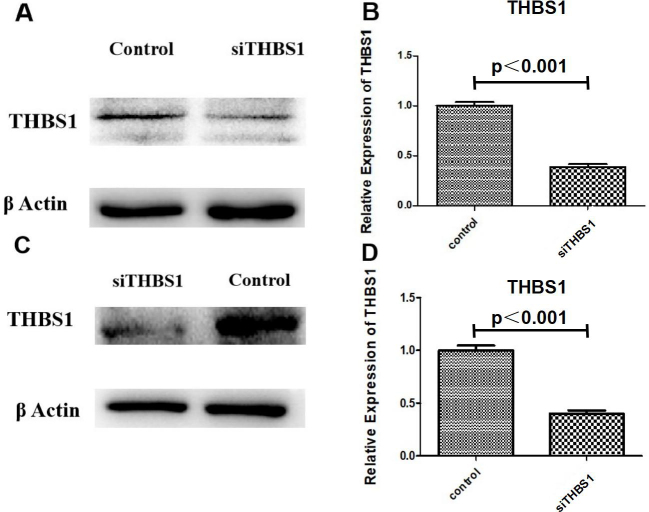
Successful knockdown of THBS1 by siRNA *in vivo* and *in vitro.* THBS1 expression was reduced in the *in vivo* model mice injected with siTHBS1, as determined using (A) western blotting and (B) RT-qPCR (significance was found in parts B between the groups). THBS1 expression was reduced in the *in vitro* primary myocardial cell cultures following siTHBS1 transfection, as determined using (C) western blotting and (D) RT-qPCR (significance was found in parts D between the groups). RT-qPCR, reverse transcription-quantitative PCR; si, small interfering RNA; THBS1, thrombospondin-1.

